# Lessons Learned from Cross-Systems Approach to COVID-19 Pandemic Response in Juvenile Justice System, Colorado, USA

**DOI:** 10.3201/eid3013.230782

**Published:** 2024-04

**Authors:** Ashley M. Tunstall, Shannon C. O’Brien, Deborah M. Monaghan, Alexis Burakoff, Renée K. Marquardt

**Affiliations:** Colorado State University, Fort Collins, Colorado, USA (A.M. Tunstall);; Colorado Department of Human Services, Denver, Colorado, USA (A.M. Tunstall, D.M. Monaghan, R.K. Marquardt);; Colorado Department of Public Health and Environment, Denver (S.C. OʼBrien, A. Burakoff);; University of Colorado, Denver (R.K. Marquardt)

**Keywords:** juvenile justice system, COVID-19, respiratory infections, severe acute respiratory syndrome coronavirus 2, SARS-CoV-2, SARS, coronavirus disease, zoonoses, viruses, coronavirus, adolescent, pandemics, communicable diseases, correctional facilities, residential facilities, health disparities, minority and vulnerable populations, adolescent development, Colorado, United States

## Abstract

The global COVID-19 pandemic illustrates the importance of a close partnership between public health and juvenile justice systems when responding to communicable diseases. Many setting-specific obstacles must be navigated to respond effectively to limit disease transmission and negative health outcomes while maintaining necessary services for youth in confinement facilities. The response requires multidisciplinary expertise and collaboration to address unique considerations. Public health mitigation strategies must balance the risk for disease against the negative effects of restrictions. Key aspects of the COVID-19 response in the juvenile justice system of Colorado, USA, involved establishing robust communication and data reporting infrastructures, building a multidisciplinary response team, adapting existing infection prevention guidelines, and focusing on a whole-person health approach to infection prevention. We examine lessons learned and offer recommendations on pandemic emergency response planning and managing a statewide public health emergency in youth confinement settings that ensure ongoing readiness.

The beginning of the COVID-19 pandemic in early 2020 challenged youth confinement facilities in the United States to quickly integrate public health response plans into practice to protect youth and personnel from widespread infection and negative health consequences (*1*,*2*). The Colorado Department of Human Services (CDHS) and Department of Public Health and Environment in the United States recognized the need to partner closely at the outset of the COVID-19 pandemic. The Colorado Division of Youth Services (DYS) within CDHS operates 15 youth services centers that serve youths 10–20 years of age who are temporarily detained or committed to DYS legal custody by district courts statewide and have varying lengths of stay (Table 1) (*3*,*4*).

**Table 1:** Descriptive characteristics of juvenile justice system and youth populations during fiscal year 2021–2022 in case study of cross-systems approach to COVID-19 pandemic response in Colorado, USA*

Characteristics	Juvenile justice system population†			Statewide youth population‡
	Detained	Committed	Paroled	
Facilities in operation§	8	9	NA	NA
Total youth population¶	1,751	622	326	829,175
Average age, y (range)	16 (10–18)	17 (13–20)	18 (14–20)	NA
Sex				
F	521 (20.6)	19 (11.4)	32 (15.9)	402,260 (48.5)
M	2,009 (79.4)	148 (88.6)	169 (84.1)	426,682 (51.5)
Race/ethnicity				
Anglo American	1,007 (39.8)	58 (34.7)	74 (36.8)	467,963 (56.4)
Hispanic/Latinx	886 (35.0)	67 (40.1)	80 (39.8)	259,991 (31.4)
African American	534 (21.1)	37 (22.2)	42 (20.9)	33,920 (4.1)
Admission statistics				
New youths	2,530	167	201	NA
Average daily population	158.5	284.4	110.3	NA
Average length of stay	22.3 d	18.5 mo	6.7 mo	NA

*Values are no. (%) except as indicated. Descriptive terminology, including racial/ethnic categories, reflect official language of the Department of Youth Services (DYS) as of 2023. Specific definitions can be reviewed in the Terms and Definitions section of the DYS Statistical Report. NA, not applicable. †Juvenile justice system data were retrieved from the Colorado DYS Statistical Report for fiscal year 2021–2022 (*3*). ‡Colorado statewide population estimates for 2021 were retrieved from the Colorado State Demography Office (*4*). §Two facilities are multipurpose and are included in both detained and committed facility counts. ¶Youth populations were 10–20 years of age.

This case study is a companion to 2 other articles in this supplement: a national perspective on lessons learned from the COVID-19 response in correctional and detention facilities (*5*) and a case study applying COVID-19 lessons to a tuberculosis outbreak in the prison system of Washington, USA (*6*). Collectively, the articles address a critical knowledge gap regarding the experiences of confined persons during the pandemic. We describe a collaborative and robust response to COVID-19 in the juvenile justice system (JJS) in Colorado that was initiated to ensure adherence to public health risk mitigation strategies while also maximizing healthy development and well-being among a vulnerable youth population.

## Unique Features of the Juvenile Justice Population

Many unique features exist among youth in the juvenile justice system (JJS) that distinguish them from youth in the community as well as from adults in confinement settings. Youth with complex trauma are overrepresented in the justice system and have higher rates of substance abuse and mental health concerns than youth in the general population (*7*). During 2020–2022, >50% of youth in DYS centers had co-occurring needs, including formal mental health intervention services and treatment level services for substance abuse.

Although the youth population overall might be healthier and at lower risk for severe outcomes from COVID-19 than adults in confinement settings, additional historic and active life stressors can increase health disparities among confined youth compared with their community counterparts (*8*). Exposure to persistent environmental stressors in a confinement setting might negatively affect youth development and increase traumatic stress responses (*9*). In addition, although a group confinement environment provides a rehabilitative structure for youth justice settings, it also creates infection control challenges. Combined with the social and developmental needs of youth compared with adults, access to social, education, and treatment programming within an environment that is already highly restrictive must be weighed when considering quarantine and isolation protocols. When applied in a confinement setting, protocols written collectively for adults and youth might dramatically limit activities more than necessary for youth alone, creating disproportionate burden. Protocols within JJS must also consider the well-being of adult staff who typically have higher risk for severe COVID-19 disease and must balance the approach to address conflicting needs. Those challenges required rapid application of COVID-19 prevention and control protocols, a communication infrastructure across multiple levels of state government that had centralized oversight and geographically diverse locally-provided services, and a system of resource allocation to meet ongoing demands.

## Whole-Person Health Approach to Infection Prevention

In the absence of JJS-specific national guidelines (*10*), a nuanced risk-benefit analysis of infection prevention recommendations was necessary in this unique setting. Specifically, a whole-person health approach (*11*) to infection prevention was used to conceptually guide the development and ongoing consideration of protocols and strategies to manage the pandemic response.

### Guidelines

Some COVID-19 mitigation measures, such as distancing, quarantine, and isolation, have unique effects in youth confinement sites; ramifications of seclusion are known for critical development and well-being of youth. For example, mitigation measures protect against infection of both youth and staff but might also negatively affect mental health when key developmental interactions are interrupted. Many unknowns existed at the start of the pandemic, creating an immediate need to establish an information and communication structure across and within departments. The cadence of published guidance lagged, yet the developmental needs of youth required timely adjustments. In addition, existing guidance lacked the nuance to capture the unique needs within the JJS setting. For example, strict quarantine guidelines aimed at protecting adults are created according to a risk-benefit analysis that is different from that which is applied to youth. Youth have a lower overall risk for severe health consequences from infection but have a higher likelihood of negative social and developmental effects from prolonged isolation and quarantine. This shifted risk-benefit analysis reinforces the need to intentionally modify universal corrections guidance to best suit the unique needs of the youth population.

### Multidisciplinary Response Team Communication

Multidisciplinary response teams enabled real-time advocacy for diverse aspects of whole-person health within the youth services system. The resultant COVID-19 pandemic response measures were more representative of a holistic approach to health and well-being for youth and enabled more timely adjustments according to youth and staff needs. Persons within the youth services system, including CDHS medical leadership, DYS behavioral health and medical services providers and leadership, youth center security staff and administration, education leadership, dining services professionals, facilities management leadership, Colorado Department of Public Health and Environment epidemiologists, and others, were invited to convene on short notice as needed to review individual case details and determine the application of facility protocols. Although the personnel time investment for this approach was substantial, it created a systematic approach statewide and enabled feedback from critical youth and frontline staff when considering modifications.

### Youth Development Considerations

Adolescence is a time of exploration whereby normative developmental tasks include building and maintaining healthy relationships and skills to promote adaptive coping (*12*). The extended restrictions of the COVID-19 pandemic rendered young persons worldwide particularly vulnerable to the negative psychosocial effects of nonpharmaceutical interventions regardless of setting (*13*). Development and emotional maturation are dependent on life experience; stepping out of this process is not just lost time but lost capacity to attend to development demands. For youth in confinement, the effects of COVID-19 pandemic restrictions was further compounded because the confinement setting is already an environment with limited choice and autonomy. The necessity of restricting interactions disrupted programming designed to address treatment needs. To mitigate those negative effects, adjustments were made to maintain opportunities for skill building, education, and interactions with family and various stakeholders. Risk mitigation strategies comprised reallocating staffing resources and technology to deliver telehealth services and education, as well as providing virtual visitations with family members to maintain support networks.

### Resources

An essential foundational element for success in the Colorado JJS setting was adequate resource allocation for personnel time across multiple roles dedicated to health-centered policies, data and tracking systems, consultation, and equipment and supplies to adequately address needs statewide. A centralized system was required for tracking and ordering inventory across entities and ongoing monitoring as specific guidance from the Centers for Disease Control and Prevention changed. The use of high-quality masks and N95 respirators by staff working among youth with variable masking behaviors enabled more youth activity and movement. Robust testing supplies aided precision infection control decisions, minimizing restrictions. As the pandemic progressed and vaccines, therapeutics, and greater knowledge about the virus became available, a quarantine-alternative method using daily antigen testing of exposed youth enabled continuation of regular education, programming, and activities.

## Conclusions

COVID-19 exacted a large toll on whole-person health across the globe, and youth in the JJS were no exception. The pandemic response in this vulnerable population in a confinement setting required novel approaches and strengthened interdepartmental relationships with public health. The lessons learned in Colorado and resulting recommendations can inform future responses to identify priorities in preparedness activities (Table 2). Those lessons can also be applied to establishing protocols in other settings to activate adaptive response efforts, incentivize protocol adherence, and aid in a coordinated and rapid response to emerging infectious disease threats.

**Table 2:** Key lessons learned and recommendations from case study of cross-systems approach to COVID-19 pandemic response in the juvenile justice system, Colorado, USA, 2020–2023

Lessons learned	Recommendations
Critical need exists for facility-level advocacy and multidisciplinary collaboration to appropriately consider unique facility-level and individual-level requirements.	Identify diverse stakeholders to partner in decision-making.
Rapid application of response protocols requires timely communication and consultation with subject matter experts to address barriers as they arise.	Establish robust communication pathways and infrastructure for real-time expert consultations.
Youth in confinement settings require diverse services and are often more vulnerable to service disruptions.	Develop juvenile justice–specific response plans.
Risk-benefit analyses can change over time and should use a whole-person health approach.	Respond to needs by using a dynamic and holistic risk assessment strategy.
Adult staff may have divergent risk profiles and access to vaccination and therapeutics compared with youths.	Be aware of vulnerable populations and create plans to mitigate risk by using a hierarchy of controls approach (*14*).
